# Monoclonal Antibodies B38 and H4 Produced in *Nicotiana benthamiana* Neutralize SARS-CoV-2 *in vitro*

**DOI:** 10.3389/fpls.2020.589995

**Published:** 2020-11-27

**Authors:** Balamurugan Shanmugaraj, Kaewta Rattanapisit, Suwimon Manopwisedjaroen, Arunee Thitithanyanont, Waranyoo Phoolcharoen

**Affiliations:** ^1^Research Unit for Plant-produced Pharmaceuticals, Chulalongkorn University, Bangkok, Thailand; ^2^Department of Pharmacognosy and Pharmaceutical Botany, Faculty of Pharmaceutical Sciences, Chulalongkorn University, Bangkok, Thailand; ^3^Department of Microbiology, Faculty of Science, Mahidol University, Bangkok, Thailand

**Keywords:** molecular farming, transient expression, *Nicotiana benthamiana*, plant recombinant proteins, plant-produced monoclonal antibody, SARS-CoV-2, COVID-19

## Abstract

The ongoing coronavirus disease 2019 (COVID-19) outbreak caused by novel zoonotic severe acute respiratory syndrome coronavirus 2 (SARS-CoV-2) was initially reported in Wuhan city, Hubei Province of China, in late December 2019. The rapid global spread of the virus calls for the urgent development of vaccines or therapeutics for human applications to combat the coronavirus infection. Monoclonal antibodies (mAbs) have been utilized as effective therapeutics for treating various infectious diseases. In the present study, we evaluated the feasibility of plant expression system for the rapid production of recently identified therapeutically suitable human anti-SARS-CoV-2 mAbs B38 and H4. Transient co-expression of heavy-chain and light-chain sequences of both the antibodies by using plant expression geminiviral vector resulted in rapid accumulation of assembled mAbs in *Nicotiana benthamiana* leaves within 4 days post-infiltration. Furthermore, both the mAbs were purified from the plant crude extracts with single-step protein A affinity column chromatography. The expression level of mAb B38 and H4 was estimated to be 4 and 35 μg/g leaf fresh weight, respectively. Both plant-produced mAbs demonstrated specific binding to receptor binding domain (RBD) of SARS-CoV-2 and exhibited efficient virus neutralization activity *in vitro*. To the best of our knowledge, this is the first report of functional anti-SARS-CoV-2 mAbs produced in plants, which demonstrates the ability of using a plant expression system as a suitable platform for the production of effective, safe, and affordable SARS-CoV-2 mAbs to fight against the spread of this highly infectious pathogen.

## Introduction

Coronavirus (CoV) outbreaks in the last two decades cause devastating impact on the human population and global economy. The zoonotic outbreaks of severe acute respiratory syndrome coronavirus (SARS-CoV) in China and Middle East respiratory syndrome coronavirus (MERS-CoV) in Saudi Arabia on 2002–2003 and 2012, respectively are the highly pathogenic betacoronaviruses reported in the 21st century. SARS-CoV-2 has recently been added to this list, which is the causative agent for the ongoing pandemic coronavirus disease 2019 (COVID-19). SARS-CoV-2, a member of the *betacoronavirus* genus in the family *Coronaviridae*, has emerged in China in late December 2019 and causes severe fever, respiratory illness, and pneumonia (Wang et al., 2020a; Zhu et al., 2020). The virus spreads rapidly, with cases of >40 million confirmed in several countries, and has become a global public health crisis. Because of its high virulence, the virus has taken down the lives of >1 million people worldwide as of October 2020 (World Health Organization [WHO], 2020). Although many vaccine or drug candidates are in clinical trials, there are no clinically approved vaccines or targeted antiviral therapeutics available to treat coronavirus infections until now. Several efforts are in progress to develop an effective vaccine or therapeutic to prevent and treat this infection (Malla et al., 2020; Thanh Le et al., 2020).

In recent decades, monoclonal antibodies (mAbs) have been studied for its application in immunotherapies and treatments for several infectious diseases. Specific mAbs against SARS-CoV-2 are highly essential for early diagnosis and disease treatment. The potential of antibodies as efficacious anti-SARS-CoV-2 therapeutics was reported in recent studies (Cao et al., 2020; Wang et al., 2020b). mAbs specific to antigenic sites on viral surface proteins have shown therapeutic efficacy and are considered as a potential therapeutic intervention against several viral infections. Similar to SARS-CoV, the receptor binding domain (RBD) located on the surface spike (S) protein of SARS-CoV-2 binds with the functional cell receptor angiotensin-converting enzyme 2 (ACE2) in humans and mediates the viral fusion and entry into the host cells (Li et al., 2003; Shang et al., 2020). The neutralizing antibodies directed against SARS-CoV-2 mainly target the RBD of S protein, which plays an important role in virus pathogenesis and antigenicity (Poh et al., 2020; Prompetchara et al., 2020; Shanmugaraj et al., 2020c). Several research groups have characterized SARS-CoV-2 neutralizing mAbs, and more recently, two human mAbs B38 and H4 were identified by a group of researchers from China in a convalescent COVID-19 patient (Wu et al., 2020) that recognize and bind to the different RBD epitopes located on the SARS-CoV-2 virus glycoprotein, thereby preventing virus ability to bind to the host cell ACE2 receptor. These mAbs exhibited great potency and are considered as a potential prophylactic and therapeutic candidates, since they efficiently neutralize SARS-CoV-2 (Jahanshahlu and Rezaei, 2020). While these mAbs represent important candidate molecules for use as diagnostic reagents and for passive immunotherapy, effective expression platforms are needed to ensure the affordability, safety, and rapid scalability of these mAbs or any other similar candidates for clinical development.

The development of recombinant DNA technology has a major impact on biotechnology that enables the production of a wide range of biopharmaceuticals in different expression hosts. The therapeutic proteins including mAbs and their derivatives have been produced in conventional expression systems based on yeast, insect, and mammalian cells. mAbs represent a successful and fastest-growing class of biopharmaceutical products that have a greatest impact on modern medicine. Unfortunately, the mAbs produced in mammalian expression systems are prohibitively expensive, which cannot be affordable to many, especially to those who are in middle- and low-income countries (Waheed et al., 2016; Kesik-Brodacka, 2018).

In recent decades, plants are considered as a safe, cost-effective, and scalable platform for the production of recombinant proteins including vaccine antigens and mAbs (Twyman et al., 2003; Fischer and Buyel, 2020). The plant expression systems offer unique advantages compared to conventional systems in terms of low production costs, scalability, low risk of pathogen contamination, flexibility, and robustness of the system, which make them a promising platform to produce commercially valuable biopharmaceuticals (Ma et al., 2013; Tschofen et al., 2016). The importance and the role of plant-made biopharmaceuticals in the fight against SARS-CoV-2 have been quite extensively commented in recent reviews (Capell et al., 2020; Rosales-Mendoza et al., 2020; Shanmugaraj et al., 2020b). The rapid nature of plant transient expression can expedite the protein production to mitigate potential pandemics. The plant-produced recombinant mAbs have been reported as potential candidates against various viral infections including chikungunya (Iyappan et al., 2018; Hurtado et al., 2020), dengue (Dent et al., 2016), Ebola (Davey et al., 2016), HIV (Teh et al., 2014), rabies (van Dolleweerd et al., 2014), West Nile virus (He et al., 2014; Sun et al., 2017), and Zika (Diamos et al., 2019). Plant-derived recombinant proteins can be produced either by stable transformation (nucleus/chloroplast) of target gene or through transient expression *via* agroinfiltration. Every plant transformation technology has its own advantages, which can be chosen based on the nature of target protein or final product. However, the latest advancement of transient plant expression systems using viral vectors and agroinfiltration demonstrates the rapid production of recombinant proteins in large-scale biomanufacturing facilities within a few days, which make them a convenient platform for the production of rapid-response vaccines or therapeutics to tackle epidemic or pandemic situations (Gelvin, 2010; Holtz et al., 2015; Buyel et al., 2017; Buyel, 2018).

Here, in the present study, we explore the potential of a plant expression system for producing therapeutically suitable human anti-SARS-CoV-2 mAbs B38 and H4 in order to use as a diagnostic reagent or therapeutics. In this context, both the mAbs were efficiently expressed by using geminiviral vector and assembled in *Nicotiana benthamiana* leaves. The expression of recombinant mAbs was determined by Western blotting and quantified by ELISA. Further, the plant-produced mAbs was purified by single-step affinity chromatography, and antigen binding specificity was assessed. Interestingly, both the plant-produced mAbs showed neutralization efficacy but with different potency against SARS-CoV-2 *in vitro.* This study provides a proof of principle for the rapid production of SARS-CoV-2 therapeutic candidates in plants to tackle emergency situations, and the successful production of functional anti-SARS-CoV-2 mAbs in plants could address the safety, cost, and other economic issues related to mAb production in other production platforms.

## Materials and Methods

### Plant Expression Vector Construction

The institutional review board of Chulalongkorn University approved the present study protocol, and all methods were performed in accordance with the relevant guidelines and regulations. The geminiviral vector (Chen et al., 2011) used in this study was kindly provided by Hugh S. Mason, Arizona State University, United States. The amino acid sequences of the human heavy-chain (HC) and light-chain (LC) variable regions (V_H_ and V_L_) of anti-SARS-CoV-2 mAb B38 and H4 were retrieved from the previous report (Wu et al., 2020). The amino acid sequence of both the antibodies was presented in the Supplementary File. Both the antibody sequences were codon-optimized to facilitate expression in *N. benthamiana*, and the gene sequences were synthesized (Genewiz, Suzhou, China). The synthesized V_H_ and V_L_ gene fragments were fused to the corresponding sequences of codon-optimized human immunoglobulin G (IgG) constant HC (C_H_) and constant LC (C_L_) regions, respectively. The synthesized V_H_ gene was digested with *Xba*I and *Bmt*I, and the V_L_ gene was digested with *Xba*I and *Afl*II and gel eluted. Similarly, the backbone of the geminiviral plant expression vector pBYR2eK2Md (pBYK-2e) containing either C_H_ or C_L_ gene fragments was obtained by digesting with respective enzymes. The gel purified products were then ligated with T4 DNA ligase (New England Biolabs, United Kingdom) following the manufacturer’s instructions. The murine leader sequence (MGWSCIILFLVATATGVHS) was included in the amino-terminal (N-terminal) and the retention signal peptide SEKDEL was added in the carboxyl-terminal (C-terminal) of the HC and LC sequences. The resulting plasmids were transformed into *Escherichia coli* strain DH10B competent cells by heat shock method and eventually moved into *Agrobacterium tumefaciens* (GV3101) *via* electroporation. *Agrobacterium* clones were screened by PCR using gene-specific forward primer and the vector-specific reverse primer (2e-R). The list of primers used in the study was provided in Table 1. The PCR cycling conditions were as follows: initial denaturation at 94°C for 5 min followed by 30 cycles of 94°C for 30 s, 55°C for 30 s, and 72°C for 60–90 s and a final extension at 72°C for 10 min. PCR amplification was performed by using *Taq* DNA polymerase (Vivantis Technologies, Malaysia). The PCR products were observed on a 1% agarose gel. The confirmed *Agrobacterium* cells containing the recombinant plasmids were used for plant transformation.

**TABLE 1:** Sequences of the oligonucleotides used for the *E. coli*/*Agrobacterium* clone confirmation by PCR.

Name	Primer Sequence (5′-3′)
B38-VH-F	CTTGTTCAGCCTGGTGGTT
B38-VL-F	GATGACCCAGTCTCCGAG
H4-VH-F	GGTTAAGAAACCTGGCGCT
H4-VL-F	GATGACCCAGTCTCCTCTG
2e-R	GCTTTGCATTCTTGACATC

### Transient Expression of Monoclonal Antibodies B38 and H4 in *N. benthamiana* Leaves

Wild-type *N. benthamiana* plants grown under controlled conditions in the greenhouse (8-h dark/16-h light cycle) were agroinfiltrated with recombinant *A. tumefaciens* strain that contained either HC or LC. Briefly, *A. tumefaciens* harboring the plant expression vector containing either HC or LC was grown in Luria–Bertani (LB) broth supplemented with 50 mg/L kanamycin, 50 mg/L gentamycin, 50 mg/L of rifampin at 28°C overnight with continuous shaking at 200 rpm. Then, the overnight-grown culture was centrifuged at 5,000 *g* for 10 min at 28°C in a tabletop centrifuge, and the pellet was resuspended in the sterile infiltration buffer [10 mM MgSO_4_, 10 mM 2-(*N*-morpholino)ethanesulfonic acid (MES) pH 5.5] to get the final optical density (OD)_600_ of 0.2. The cell suspension containing HC and LC construct was equally mixed in a 1:1 ratio and co-infiltrated in the leaves of 6–8-week-old *N. benthamiana* plants by vacuum infiltration. The infiltrated plants were incubated in a controlled environment with high humidity at 28°C for 4 days. The agroinfiltrated leaves were harvested and processed for further analysis.

### Protein Extraction and Purification

To purify anti-SARS-CoV-2 mAb (B38 and H4) from plants, the infiltrated plant leaves were harvested 4 days post infiltration (dpi) and purified by protein A column chromatography as described previously (Rattanapisit et al., 2019a). Briefly, the harvested leaves were homogenized with extraction buffer 1 × phosphate-buffered saline (PBS; 137 mM NaCl, 2.7 mM KCl, 4.3 mM Na_2_HPO_4_, 1.47 mM KH_2_PO_4_) at a ratio of 1 g leaf tissue to 2 ml buffer, and the plant crude extracts were centrifuged at 26,000 *g* for 40 min at 4°C. After centrifugation, supernatant was collected and filtered through 0.45-μm membrane filter. Amintra protein A resin (Expedeon, United Kingdom) was packed in the column and equilibrated with 1 × PBS (pH 7.4). After equilibration, the recombinant mAbs in the filtered protein extract was loaded into a protein A bead column. The column was further washed with 15 column volumes of 1 × PBS, and the bound protein was eluted using elution buffer (0.1 M glycine, pH 2.7) and neutralized with 1.5 M Tris-HCl (pH 8.8). Following the purification, the eluted fractions were analyzed on sodium dodecyl sulfate-polyacrylamide gel electrophoresis (SDS-PAGE) gel stained with Coomassie brilliant blue (AppliChem, Germany) and Western blotting.

### Sodium Dodecyl Sulfate-Polyacrylamide Gel Electrophoresis and Western Blotting

The plant-produced mAbs were mixed with non-reducing sample buffer [125 mM Tris-HCl pH 6.8, 12% (w/v) SDS, 10% (v/v) glycerol, 0.001% (w/v) bromophenol blue] and separated on 6%–15% polyacrylamide gel as described previously (Rattanapisit et al., 2019b). For reducing conditions, 22% (v/v) β-mercaptoethanol was added in the sample buffer and separated on 6–15% polyacrylamide gel. The protein samples were boiled at 95°C for 5 min and separated in the gel for 90 min at 100 V. All blue prestained protein standards (Bio-Rad, United States) were included in SDS-PAGE and Western blots as protein molecular weight markers. After electrophoresis, gels were stained with Coomassie brilliant blue, and the protein bands were visualized. For Western blotting, the protein in the gel was electro-transferred on to 0.45-μm nitrocellulose membrane (Bio-Rad, United States) for 90 min at 100 V. The membrane was blocked with 5% skim milk in 1 × PBS for 1 h at room temperature, washed with PBST (PBS with 0.05% Tween-20) thrice for 5 min each, and probed with either anti-human gamma antibody (The Binding Site, United Kingdom) or anti-human kappa antibody conjugated with horseradish peroxidase (HRP) (Southern Biotech, United States) diluted 1:5,000 in 3% skim milk in 1 × PBS. After incubation for 2 h, the plates were washed with PBST thrice, and the bound antibody was detected by chemiluminescence using ECL plus detection reagent (GE Healthcare, United Kingdom) in accordance with the manufacturer’s procedures.

### Monoclonal Antibody Expression Level Quantification by ELISA

The expression level of plant-produced mAbs was determined by ELISA using commercially available human IgG1 kappa isotype antibody (Abcam, United Kingdom) as standard. Technical triplicates were assayed for all the samples and standard. Briefly, the 96-well plates were coated with anti-human IgG-Fc fragment specific (Abcam, United Kingdom) and incubated overnight at 4°C. Then, the plates were washed with PBST for three times and blocked with 5% skim milk powder in 1 × PBS for 2 h at 37°C. After being washed with PBST, the dilutions of plant crude extracts and standard were added to the plate. After 2-h incubation, the plate was washed and HRP-conjugated anti-human kappa antibody (Southern Biotech, United States) with a dilution of 1:1,000 in 1 × PBS was added and incubated for 1 h at 37°C. The plate was then washed three times with PBST, developed with 3,3′,5,5′-tetramethylbenzidine (TMB) substrate (Promega, United States). The reaction was stopped by adding 1 M H_2_SO_4_, and absorbance was measured at 450 nm.

### Binding Efficiency of Plant-Produced Monoclonal Antibody to SARS-CoV-2 Receptor Binding Domain Protein

ELISA to examine the binding of mAb B38 and H4 to SARS-CoV-2 RBD protein was performed. Three biological replicates were performed, and each sample was subjected to three technical replicates to assess the mAb binding efficiency. Briefly, RBD of SARS-CoV-2 protein (His tagged RBD produced in Sf9 insect cells, Z03479; Genscript Biotech, United States) was bound to 96-well microplates (Greiner Bio-One GmbH, Frickenhausen, Germany). After overnight incubation, the plates were blocked with 5% skim milk (BD, Franklin Lakes, NJ, United States) in 1 × PBS for 2 h, washed three times with PBST, and incubated with plant-produced mAbs B38 and H4. After 2-h incubation at 37°C, sheep anti-human kappa LC conjugated with HRP (Southern Biotech, United States) at a dilution of 1:1,000 in 1 × PBS was added, and samples were incubated for 1 h at 37°C. The plate was then washed three times with PBST, developed using TMB substrate (Promega, United States), and the absorbance was read at 450 nm. The commercially available human IgG1 (Abcam, United Kingdom) and plant-produced human anti-PD1 antibody (Rattanapisit et al., 2019b) was used as negative controls.

### Severe Acute Respiratory Syndrome Coronavirus 2 Neutralization

All the experiments with live SARS-CoV-2 virus were performed at a certified biosafety level 3 facility, Department of Microbiology, Faculty of Science, Mahidol University, Thailand. The experimental protocol was approved by Mahidol University, and all methods were performed by following standard protocols approved by the institutional review committee. In this study, we used SARS-CoV-2 virus (SARS-CoV-2/01/human/Jan2020/Thailand) isolated from a confirmed COVID-19 patient at Bamrasnaradura Infectious Diseases Institute, Nonthaburi, Thailand, for *in vitro* experiments. Positive convalescent serum collected from a different COVID-19 patient was approved to be used as a clinical specimen by the Faculty of Medicine, Ramathibodi Hospital. The informed consent was waived by the institutional review boards that approved the present study.

Briefly, the plant-produced mAbs B38 or H4 or positive control or negative control was twofold serially diluted in 96-well plates. Then, 100 × TCID_50_ (50% tissue culture infectious dose) of the SARS-CoV-2 virus was added to the mAb dilutions and incubated for 1 h at 37°C. The Vero-E6 cells pre-seeded in Dulbecco’s modified Eagle’s medium (DMEM) supplemented with 2% fetal bovine serum (FBS), 100 U/ml of penicillin, and 0.1 mg/ml of streptomycin (1 × 10^4^ cells/well, duplicates) were then infected with the mAb–virus mixture for 2 days. The cells were then fixed and permeablized with ice-cold 1:1 methanol/acetone fixative for 20 min at 4°C. The fixed plates were washed three times with 1 × PBS containing 0.05% Tween 20 (wash buffer) and blocked with blocking buffer containing 2% bovine serum albumin (BSA) and 0.1% tween 20 in 1 × PBS for 1 h. After washing the plates thrice with wash buffer, the SARS-CoV/SARS-CoV-2 nucleocapsid mAb (40143-R001; Sino Biological, United States) with a dilution of 1:5,000 in 1 × PBS containing 0.5% BSA and 0.1% Tween 20 was added to each well and incubated for 2 h at 37°C. The plates were then washed three times with wash buffer, and HRP-conjugated goat anti-rabbit polyclonal antibody (P0448; Dako, Denmark) was used as secondary antibody at a dilution 1:2,000. After incubation for 1 h at 37°C, the plates were washed thrice and detected with SureBlue TMB 1 component microwell peroxidase substrate (SeraCare Life Sciences, United States). The reaction was stopped with 1N HCl. The absorbance was measured at 450 and 620 nm (reference wavelength) with an ELISA plate reader. Both virus control (no antibody) and cell control (no virus, no antibody) were included in the plates. Convalescent serum collected from the COVID-19 patient (heat inactivated at 56°C, 30 min) was used as a positive control; anti-human PD1 antibody (Rattanapisit et al., 2019b) and negative serum were used as negative controls. The experiment was performed with three technical replicates for each sample. The average ODs at 450 and 620 nm were determined for virus control and cell control wells, and the neutralizing endpoint was determined by 50% specific signal calculation. The neutralizing endpoint titer was expressed as the reciprocal of the highest serum dilution with an OD value less than X (Rowe et al., 1999; World Health Organization [WHO], 2011), which was calculated as follows:

$$\begin{array}{c} X=[(\mathrm{average A450}-\mathrm{A620 of}\;100\times {\mathrm{TCID}}_{50}\;\mathrm{virus control wells})\\ \;-\mathrm{average A450}-\mathrm{A620 of cell control wells})]/2\\ \;+\mathrm{average A450}-\mathrm{A620 of cell control wells}). \end{array}$$

Sera which tested negative at 1:10 dilution were assigned a titer of <10. Sera were considered positive if neutralization titer is ≥20.

## Results

### Expression of Monoclonal Antibodies Using Geminiviral Plant Expression Vector

**FIGURE 1:**
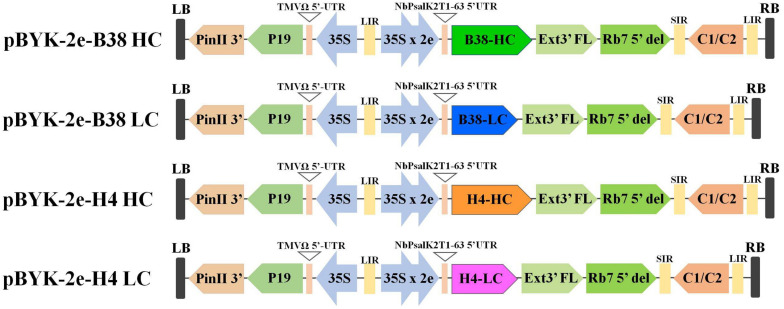
Generalized schematic representation of the monoclonal antibodies (mAbs) B38 and H4 heavy chain (HC) and light chain (LC) in plant expression constructs used in the present study. RB and LB, the right and left borders of the T-DNA region transferred by *Agrobacterium* into plant cells; 35S, Cauliflower Mosaic Virus (CaMV) 35S promoter; 35S x 2e, CaMV 35S promoter with duplicated enhancer; NbPsalK2T1-63 5′UTR, 5′ untranslated region; B38-HC, HC of B38 antibody; B38-LC, LC of B38 antibody; H4-HC, HC of H4 antibody; H4-LC, LC of H4 antibody; Ext3′FL, 3′ region of tobacco extension gene; Rb7 5’ del, tobacco RB7 promoter; SIR, short intergenic region of BeYDV; LIR, long intergenic region of BeYDV; C2/C1, Bean Yellow Dwarf Virus (BeYDV) ORFs C1 and C2 encoding for replication initiation protein (Rep) and RepA; TMVΩ 5′-UTR, 5′ untranslated region of tobacco mosaic virus Ω; P19, the RNA silencing suppressor from tomato bushy stunt virus; PinII 3′, the terminator from potato proteinase inhibitor II gene.

**FIGURE 2:**
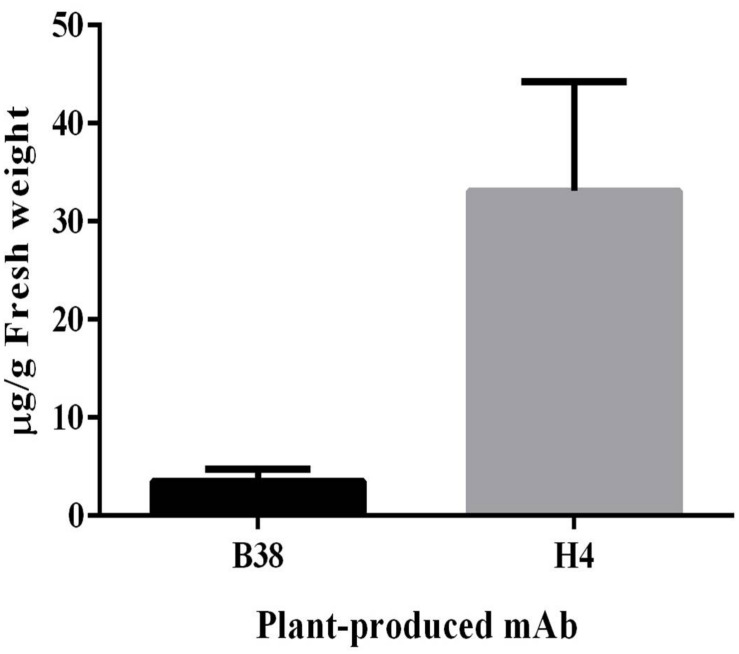
Expression levels of plant-produced monoclonal antibodies (mAbs) B38 and H4 were measured by ELISA. *N. benthamiana* leaves agroinfiltrated with heavy-chain (HC) and light-chain (LC) constructs were harvested at 4 days post infiltration (dpi), and the expression was analyzed using human immunoglobulin G (IgG) as standard. The result was presented on the leaf fresh weight (FW) basis. The data are expressed as mean ± standard deviation of three independent replicates.

The expression of mAbs B38 and H4 in *N. benthamiana* was accomplished by cloning codon-optimized HC and LC sequences into the geminiviral vector pBYK-2e to create the expression cassettes pBYK-2e-B38 HC, pBYK-2e-B38 LC, pBYK-2e-H4 HC, and pBYK-2e-H4 LC. The coding sequences of the antibody genes were incorporated into the vector under the control of the CaMV 35S promoter with duplicated enhancer to drive maximal transcription, and the RNA silencing suppressor P19 was also present in the vector for enhanced protein production (Diamos and Mason, 2019). The generalized schematic diagram of the T-DNA regions of the plant expression vectors used in the present study was shown in Figure 1. The LC and HC expression cassette was co-infiltrated into the leaves of wild-type *N. benthamiana* by vacuum agroinfiltration. The protein extracts were collected from the agroinfiltrated leaves 4 dpi, and the recombinant mAbs were purified. The assembled mAbs produced from *N. benthamiana* were quantified by ELISA. The expression levels of mAbs B38 and H4 were estimated to be 4 and 35 μg/g fresh weight (FW), respectively (Figure 2).

### Purification of Recombinant Monoclonal Antibodies From Plant Crude Extract

**FIGURE 3:**
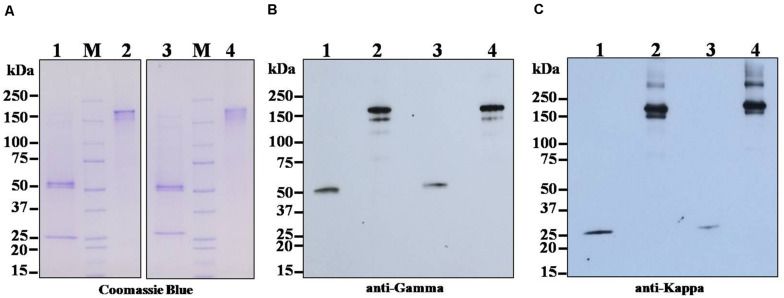
Sodium dodecyl sulfate-polyacrylamide gel electrophoresis (SDS-PAGE) and Western blot analysis of transiently expressed monoclonal antibodies (mAbs) B38 and H4 in *N. benthamiana* leaves. *Agrobacterium* containing the plant expression geminiviral cassette with heavy chain (HC) or light chain (LC) of the antibody was co-infiltrated into the *N. benthamiana* leaves. The leaf samples were harvested 4 days post infiltration (dpi), and protein extracts were separated by SDS-PAGE and stained with Coomassie blue stain **(A)**. For Western blot, the protein was electroblot transferred onto a nitrocellulose membrane, and the membrane was probed with either HRP-conjugated anti-human gamma chain antibody **(B)** or anti-human kappa chain antibody **(C)**. Lane 1: Plant-produced mAb B38 under reducing condition; Lane 2: Plant-produced mAb B38 under non-reducing condition; Lane 3: Plant-produced mAb H4 under reducing condition; Lane 4: Plant-produced mAb H4 under non-reducing condition.

The anti-SARS-CoV-2 mAbs (B38 and H4) from the leaf crude extract of *N. benthamiana* plants were purified by using protein A affinity column chromatography. The identity of the purified mAbs was confirmed by SDS-PAGE and Western blotting. The separated bands in the SDS-PAGE gel was stained with Coomassie brilliant blue stain, and the bands were visualized. For Western blotting, the separated proteins were transferred to the nitrocellulose membrane and detected with HRP-conjugated anti-human gamma chain antibody or anti-human kappa chain antibody. As shown in Figure 3, HC and LC bands of both the mAbs were observed with the expected molecular size of approximately 150 kDa under non-reducing conditions (Figures 3A–C, Lanes 2 and 4), which is possibly the heterotetramer of the fully assembled antibody (mAb B38 and H4). Some additional molecular weight bands are also observed, probably indicating the assembly intermediates or proteolytic fragments (Hehle et al., 2011; van Dolleweerd et al., 2014). Under reducing conditions, predominant ∼50 kDa band was typically visible in Coomassie-stained SDS-PAGE gel (Figure 3A, Lanes 1 and 3) and Western blot probed with HRP-conjugated anti-human gamma chain antibody corresponding to the expected size for HC (Figure 3B, Lanes 1 and 3) of the antibody, whereas ∼25 kDa band was visible in Coomassie-stained SDS-PAGE gel (Figure 3A, Lanes 1 and 3) and Western blot probed with HRP-conjugated anti-human kappa chain antibody (Figure 3C, Lanes 1 and 3), which corresponds to LC of both the plant-produced antibodies. This result confirms the co-expression of HC and LC resulting in the expression of fully assembled anti-SARS-CoV-2 mAbs B38 and H4 in *N. benthamiana*.

### Recognition of Plant-Produced Monoclonal Antibody to SARS-CoV-2 Receptor Binding Domain Protein

**FIGURE 4:**
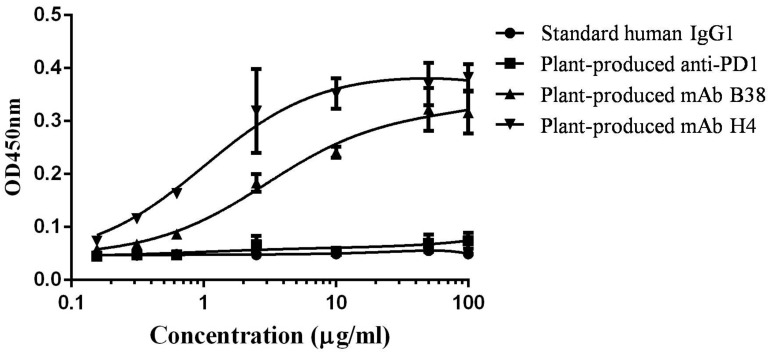
Binding of plant-produced monoclonal antibodies (mAbs) B38 and H4 to severe acute respiratory syndrome coronavirus 2 (SARS-CoV-2) receptor binding domain (RBD) protein. The ability of the plant-produced mAbs to recognize RBD protein of SARS-CoV-2 was assessed by ELISA. The plant-produced mAbs B38 and H4, standard human immunoglobulin G (IgG)1, and plant-produced anti- PD1 antibody (as negative control) (Rattanapisit et al., 2019b) were incubated on plates coated with commercial SARS-CoV-2 RBD. The bound antibody was detected with a horseradish peroxidase (HRP)-conjugated anti-human kappa antibody. The data are the mean values of technical triplicates per concentration.

Since both the human mAbs H4 and B38 were reported to recognize different epitopes on RBD domain of S protein on SARS-CoV-2, it was indispensable to show that the plant-produced recombinant mAb versions have the ability to recognize and bind the RBD domain of SARS-CoV-2. ELISA was performed in which SARS-CoV-2 RBD was coated on 96-well microplates followed by adding serial dilutions of purified recombinant mAbs B38 and H4. As shown in Figure 4, the plant-produced antibody recognized the RBD protein, whereas negligible reactivity was observed in the negative controls. Together, these data suggested that the plant-produced recombinant antibodies have the ability to bind the RBD protein of SARS-CoV-2.

### Neutralization Activity of Monoclonal Antibody Against SARS-CoV-2

The neutralization potential of plant-produced mAbs B38 and H4 against SARS-CoV-2 was evaluated. The detection of nucleocapsid protein indicates virus infection and lack of neutralizing antibodies. The results (Table 2) show that the plant-produced antibodies B38 exhibit a neutralization titer of 40 at the concentration of 5.45 μg/ml, and H4 showed a neutralization titer of 640 at the concentration of 0.492 μg/ml against SARS-CoV-2 *in vitro*, while the negative controls, i.e., anti-human PD1 antibody and negative serum (<10) did not neutralize SARS-CoV-2. The neutralizing titer of plant-derived mAb H4 was superior to that of plant-derived mAb B38. These results proved that the anti-SARS-CoV-2 mAbs produced in *N. benthamiana* are functional and exhibited neutralizing activity against highly infectious SARS-CoV-2.

**TABLE 2:** The neutralizing activity of plant-produced B38 and H4 monoclonal antibodies (mAbs) against severe acute respiratory syndrome coronavirus 2 (SARS-CoV-2).

Antibody	Neutralization titer^a^
B38	40
H4	640
Positive serum	2560
Negative serum	< 10
Anti-human PD1 antibody	< 10

*The experiment was performed with three technical replicates for each sample. Dilutions of plant-produced mAbs or serum collected from a coronavirus disease 2019 (COVID-19) patient (positive control) or human anti-PD1 antibody, negative serum (negative control) were co-incubated with 100 × TCID_50_ of the SARS-CoV2 virus and Vero-E6 cells for 48 h, and then neutralizing titers of the mAbs were calculated. ^a^Titer of <10 indicates no detectable antibody and considered negative.*

## Discussion

The unprecedented speed spread of the SARS-CoV-2 is responsible for the current pandemic, and the explosion of the number of COVID-19 cases associated with increasing mortality rates demands for effective diagnosis and treatment. Although many groups are working toward developing a COVID-19 vaccine, there is an equally vital competition going on parallelly to identify and produce potential targeted mAbs that could provide an immediate boost to the immune system to fight against the virus. Indeed, human mAbs targeting against S glycoprotein of SARS-CoV-2 have been shown to be protective against SARS-CoV-2 infection (Pinto et al., 2020; Wang et al., 2020b; Wec et al., 2020). The recently identified human neutralizing antibodies B38 and H4 shown to reduce virus titers in animal models are considered potential candidates to use either alone or in combination with other mAbs to treat COVID-19 and also possibly other future coronavirus infections (Wu et al., 2020). These findings suggest the utility of the neutralizing antibodies as a promising prophylactic or therapeutic candidate against SARS-CoV-2. However, the high production cost of therapeutic mAbs in conventional expression systems might limit their use in developing and underdeveloped countries (Kelley, 2009). The global pandemic highlights the need for expression platforms that can bring the therapeutic mAbs to the market with rapid production speed, mass production, and affordability.

The production of biopharmaceutical proteins in plants has received much attention in recent years, and several proteins with commercial, industrial, or pharmaceutical applications have been produced by plant cells, tissues, or whole plants (Tschofen et al., 2016; Donini and Marusic, 2019). Remarkably, the US Food and Drug Administration (FDA) approval of the first plant-made pharmaceutical Elelyso^TM^ (taliglucerase alfa) for the treatment of Gaucher’s disease (Fox, 2012) and first in-human clinical trial of the transgenic tobacco-derived anti-HIV antibody 2G12 (Ma et al., 2015) made a significant breakthrough in the field of plant molecular farming that has established the regulatory framework and opens the way for large pharmaceutical companies to manufacture plant-made pharmaceuticals. Further, the rapid nature of the transient expression system was well demonstrated during the Ebola outbreak by Mapp Biopharmaceutical Inc. (Davey et al., 2016). In addition, extensive work has been done to optimize and express various vaccine antigens and therapeutic antibodies in plant expression systems for the diagnosis, prevention, and treatment of several infectious diseases (Shahid and Daniell, 2016; Zhang et al., 2017; Massa et al., 2018; Schillberg et al., 2019; Rybicki, 2020; Shanmugaraj et al., 2020a).

In this study, we demonstrated the production and efficacy of two candidate SARS-CoV-2 mAbs B38 and H4 in *N. benthamiana* in order to develop affordable antibody-based therapeutics against SARS-CoV-2. The nucleotide sequences of both the mAbs were codon-optimized to boost the recombinant protein expression in plants. The plant expression geminiviral vector was used for the transient expression of both the mAbs in *N. benthamiana*. Earlier reports have shown that the transient *Agrobacterium*-mediated transformation with geminiviral replicon vector derived from bean yellow dwarf virus produces mAbs in tobacco (Chen et al., 2011; Diamos and Mason, 2019). Our results showed that both mAbs B38 and H4 accumulated efficiently in *N. benthamiana* leaves within 4 dpi at a level of 4 and 35 μg/g FW, respectively. Transient expression offers several advantages and is the method of choice to manufacture high levels of proteins to meet the unforeseen demand of recombinant viral proteins or therapeutic antibodies during epidemic situations. The striking feature of the plant transient expression system is that it requires a few weeks to produce recombinant proteins after constructing the plant expression vector. Hence, it is considered as a promising alternative to the generation of stable transformants by transgenic approach, which usually takes 3–6 months (Peyret and Lomonossoff, 2015; Yao et al., 2015; Rybicki, 2017).

Further, the antigen binding ability of purified mAbs B38 and H4 to SARS-CoV-2 RBD was determined. ELISA results showed that plant-produced anti-SARS-CoV-2 mAbs can bind to the RBD protein of SARS-CoV-2, whereas no binding was observed in the negative control. Hence, it was confirmed that the mAbs were correctly assembled in the plant system and retained its ability to bind to RBD protein of SARS-CoV-2. The neutralizing activity of the plant-produced mAbs was also investigated *in vitro*, and the results demonstrated that both the plant-produced antibodies have a neutralizing activity against SARS-CoV-2, highlighting the potency of plant-produced anti-SARS-CoV-2 mAbs. Wu et al. (2020) reported that HEK-293T-produced antibodies B38 and H4 exhibited a neutralizing activity in Vero E6 cells and protective efficacy in a mouse model. In either case, B38 was shown to be effective compared to H4, whereas our results showed that plant-produced H4 was found to be better than B38 in RBD binding and neutralizing SARS-CoV-2 *in vitro*. Although the cause of this disparity is unclear at this moment, we speculate that both B38 and H4 produced in wild-type *N. benthamiana* carried plant-specific glycans that might have an impact on the antigen binding and biological function of plant-produced mAbs (Ko et al., 2003; Miranda et al., 2007; Scallon et al., 2007; Strasser et al., 2009). One way of circumventing the difference in the complex glycans between mammals and plants is to use gycoengineered plants for the production of structurally similar mAbs as those produced in mammalian platforms, which could improve the therapeutic utility of mAbs (Schahs et al., 2007; Strasser et al., 2008; Montero-Morales and Steinkellner, 2018; Sukenik et al., 2018). In this way, anti-SARS-CoV-2 mAbs can be produced with more homogeneous human-like glycans, and its efficacy can be compared with mammalian counterparts. Available evidence suggests that some of the plant-produced recombinant proteins exhibit equivalent or better activity compared to their mammalian-produced counterparts *in vitro* and *in vivo* (Zeitlin et al., 2011; Olinger et al., 2012; Lai et al., 2014).

Our group has previously reported that the anti-SARS-CoV neutralizing antibody CR3022 (ter Meulen et al., 2006) can be efficiently produced in plants, which could be used as a diagnostic reagent for SARS-CoV-2 detection. In contrary to the present results, the plant-produced CR3022 can effectively bind with RBD of SARS-CoV-2 but did not neutralize SARS-CoV-2 *in vitro*, which could be due to the fact that epitope of CR3022 does not overlap with the ACE2 binding site (Rattanapisit et al., 2020; Tian et al., 2020). Unlike CR3022, mAbs B38 and H4 exhibit competition with ACE2 for RBD binding and recognize different epitopes on the RBD of SARS-CoV-2, allowing them to effectively neutralize SARS-CoV-2 by preventing the virus binding with the cellular receptor ACE2 (Wu et al., 2020). Altogether, the results from antigen binding specificity and neutralization ability demonstrate that the plant-produced mAbs B38 and H4 were properly folded, fully functional, and retained their biological activity after purification.

In order to effectively control the spread of the infection, a therapeutic approach based on mAbs should provide sufficient breadth of protection against different strains of SARS-CoV-2. In such cases, a single mAb might not be sufficient to prevent viral escape and extend the breadth of protection. Hence, the application of two or more mAb cocktails for effective passive immunoprophylaxis is needed, which requires a cost-effective platform to manufacture such mAbs, so that it can be accessible outside the developed world. The successful demonstration of rapid production of mAbs B38 and H4 in plants might help to address the safety and cost-related issues of immunotherapeutics, since earlier studies have shown that the plant recombinant proteins can be produced with significant reduction in manufacturing costs and capital expenditures. Further plant-based production of biologics can reduce the upstream production cost to $1.00–$2.00 per kg of protein, which can ensure equitable access to affordable therapeutics. However, a high level of recombinant protein expression in plant expression systems and product recovery warrants the future success of the plant-derived biopharmaceuticals (Chen, 2008; Tuse et al., 2014; Chen and Davis, 2016; Nandi et al., 2016; McNulty et al., 2020). Indeed, a detailed techno-economic assessment of the economic significance and rapid nature of plant system is critical to estimate the production cost of anti-SARS-CoV-2 mAbs in *N. benthamiana* at the commercial scale. In summary, we demonstrated the rapid production, purification, and protective efficacy of two therapeutic candidate mAbs B38 and H4 in *N. benthamiana.* To our knowledge, this is the first report of the plant-produced mAb that neutralizes SARS-CoV-2 *in vitro*. However, safety, *in vivo* breadth, and potency studies in animal models warrant the efficacy of these plant-produced mAbs toward clinical development. Taken together, our results may lead as a proof of principle study that can readily be applied to generate safer antibody-based therapeutics against SARS-CoV-2 or other emerging infectious disease threats.

## Data Availability Statement

The original contributions presented in the study are included in the article/Supplementary Material, further inquiries can be directed to the corresponding author.

## Ethics Statement

The studies involving human participants were reviewed and approved by Human Research Ethics Committee, Faculty of Medicine Ramathibodi Hospital, Mahidol University. Written informed consent for participation was not required for this study in accordance with the national legislation and the institutional requirements.

## Author Contributions

WP and AT conceived and designed the experiments. BS and KR constructed the plant expression vectors and performed the protein expression, protein purification, and antigen binding by ELISA. SM and AT performed the *in vitro* neutralization experiment. BS, WP, and AT analyzed the data and wrote the manuscript. All the authors read and approved the final version of the manuscript.

## Conflict of Interest

WP from Chulalongkorn University is a founder/shareholder of Baiya Phytopharm Co., Ltd. The remaining authors declare that the research was conducted in the absence of any commercial or financial relationships that could be construed as a potential conflict of interest.
