# Supplementary material for: Monoclonal Antibodies B38 and H4 Produced in Nicotiana benthamiana Neutralize SARS-CoV-2 in vitro
Source: Front Plant Sci. 2020 Nov 27;11:589995. doi: 10.3389/fpls.2020.589995 (PMC7728718; doi:10.3389/fpls.2020.589995)
Supplement: Supplementary file 1 [file Table_1.DOC]

Supplementary Information

**Monoclonal Antibodies B38 and H4 Produced in *Nicotiana benthamiana* Neutralize SARS-CoV-2 *in vitro***

Balamurugan Shanmugaraj^1,2,§^, Kaewta Rattanapisit^1,2,§^, Suwimon Manopwisedjaroen^3^, Arunee Thitithanyanont^3^, Waranyoo Phoolcharoen^1, 2^*

The amino acid sequences of mAb B38 and H4 (Wu et al., 2020) are listed below.

**B38-HC:**

EVQLVESGGGLVQPGGSLRLSCAASGFIVSSNYMSWVRQAPGKGLEWVSVIYSGGSTYYADSVKGRFTISRHNSKNTLYLQMNSLRAEDTAVYYCAREAYGMDVWGQGTTVTVSSASTKGPSVFPLAPSSKSTSGGTAALGCLVKDYFPEPVTVSWNSGALTSGVHTFPAVLQSSGLYSLSSVVTVPSSSLGTQTYICNVNHKPSNTKVDKKVEPKSCDKTHTCPPCPAPELLGGPSVFLFPPKPKDTLMISRTPEVTCVVVDVSHEDPEVKFNWYVDGVEVHNAKTKPREEQYNSTYRVVSVLTVLHQDWLNGKEYKCKVSNKALPAPIEKTISKAKGQPREPQVYTLPPSRDELTKNQVSLTCLVKGFYPSDIAVEWESNGQPENNYKTTPPVLDSDGSFFLYSKLTVDKSRWQQGNVFSCSVMHEALHNHYTQKSLSLSPGK

**B38-LC:**

DIVMTQSPSFLSASVGDRVTITCRASQGISSYLAWYQQKPGKAPKLLIYAASTLQSGVPSRFSGSGSGTEFTLTISSLQPEDFATYYCQQLNSYPPYTFGQGTKLEIKTVAAPSVFIFPPSDEQLKSGTASVVCLLNNFYPREAKVQWKVDNALQSGNSQESVTEQDSKDSTYSLSSTLTLSKADYEKHKVYACEVTHQGLSSPVTKSFNRGEC

**H4-HC:**

QVQLVQSGAEVKKPGASVKVSCKASGYTFTGYYMHWVRQAPGQGLEWMGRINPNSGGTNYAQKFQGRVTMTRDTSISTAYMELSRLRSDDTAVYYCARVPYCSSTSCHRDWYFDLWGRGTLVTVSSASTKGPSVFPLAPSSKSTSGGTAALGCLVKDYFPEPVTVSWNSGALTSGVHTFPAVLQSSGLYSLSSVVTVPSSSLGTQTYICNVNHKPSNTKVDKKVEPKSCDKTHTCPPCPAPELLGGPSVFLFPPKPKDTLMISRTPEVTCVVVDVSHEDPEVKFNWYVDGVEVHNAKTKPREEQYNSTYRVVSVLTVLHQDWLNGKEYKCKVSNKALPAPIEKTISKAKGQPREPQVYTLPPSRDELTKNQVSLTCLVKGFYPSDIAVEWESNGQPENNYKTTPPVLDSDGSFFLYSKLTVDKSRWQQGNVFSCSVMHEALHNHYTQKSLSLSPGK

**H4-LC:**

DIQMTQSPLSLPVTPGEPASISCRSSQSLLDSDDGNTYLDWYLQKPGQSPQLLIYTLSYRASGVPDRFSGSGSGTDFTLKISRVEAEDVGVYYCMQRIEFPLTFGGGTKVEIKTVAAPSVFIFPPSDEQLKSGTASVVCLLNNFYPREAKVQWKVDNALQSGNSQESVTEQDSKDSTYSLSSTLTLSKADYEKHKVYACEVTHQGLSSPVTKSFNRGEC

**Reference**

Wu, Y., Wang, F., Shen, C., Peng, W., Li, D., Zhao, C., et al. (2020). A noncompeting pair of human neutralizing antibodies block COVID-19 virus binding to its receptor ACE2. *Science* 368(6496)**,** 1274-1278. doi: 10.1126/science.abc2241.
